# Effects of Lactic Acid Bacteria from Pickles on the Silage Fermentation and Bacterial Community and Anerobic Stability of Maize, Soybean and Their Mixture in Karst Regions

**DOI:** 10.3390/microorganisms14030528

**Published:** 2026-02-25

**Authors:** Yujia Wang, Xiaokang Huang, Chaosheng Liao, Xiaolong Tang, Tu Hong, Yubo Zhang, Pan Wang, Chao Chen, Ping Li

**Affiliations:** 1 College of Animal Science, Guizhou University, Guiyang 550025, China; 17785844823@163.com (Y.W.); h15329727872@163.com (X.H.); liaocs@yeah.net (C.L.); txl971217@sina.com (X.T.); qq583712918@gmail.com (T.H.); zyb0129gz@sina.cn (Y.Z.); 18685204997@163.com (P.W.); 2 Key Laboratory of Animal Genetics and Breeding & Reproduction in the Plateau Mountainous Region, Inistry of Education, Guizhou University, Guiyang 550025, China

**Keywords:** silage, lactic acid bacteria, pickles, fermentation, karst region

## Abstract

This study aims to investigate the effects of three lactic acid bacteria (LAB) strains, *Lactiplantibacillus plantarum*, *Lactiplantibacillus pentosus* and *Limosilactobacillus fermentum*, isolated from traditional pickles in Guizhou, on the fermentation process and microbial community dynamics of ensiled whole-plant maize, soybean, and their mixtures. The results revealed that compared to the CK group, the lactic acid levels of *Lactiplantibacillus plantarum* and *Lactiplantibacillus pentosus* were significantly increased in the treatment groups (*p* < 0.05), resulting in a faster pH reduction, along with decreases in ammonia nitrogen (AN) and butyric acid (BA) content. In contrast, the *Limosilactobacillus fermentum* treatment (*p* < 0.05) promoted acetic acid (AA) production and inhibited the growth of harmful microbiota in soybean silage. Notably, inoculation with all LAB strains enhanced the aerobic stability of maize silage by promoting the proliferation of *Lactiplantibacillus* during the later stages of fermentation, thereby sustaining a low pH and mitigating the depletion of water-soluble carbohydrates (WSC). Furthermore, all treatments accelerated silage fermentation by enhancing the LAB population and competing with yeast and *Escherichia coli* for available nutrients in mixed silage. These findings indicate that three LAB strains, when used as microbial additives, demonstrated potential to improve silage quality in the Karst region.

## 1. Introduction

With advancements in modern microbiome technologies, the application of LAB has extended beyond traditional food fermentation, increasingly finding use in the animal feed industry [1]. The LAB is widely regarded as a probiotic conferring multiple health benefits, including maintaining intestinal microecological balance through competitive exclusion of pathogenic microbiota, enhancement of immune function, and improvement of nutrient absorption [2]. In biorefining and animal husbandry, applying LAB inoculants for silage preservation is widely recognized as a reliable and efficient method for maintaining the freshness of forage feedstock [3]. The biochemical transformations during silage fermentation stabilize the material and stimulate research into novel silage additives. Due to their dominant role in driving anaerobic fermentation and inhibiting spoilage organisms, the LAB are pivotal in ensuring silage quality, underscoring the potential of specific strains to achieve superior biopreservation performance [4]. Consequently, the LAB strains are increasingly utilized as versatile microbial additives in crop and feed biomass [5].

Notably, during the fermentation of plant biomass, the LAB synthesize lactic acid and other beneficial organic acids, which collectively contribute to reducing pH and inhibiting spoilage microbiota. These biochemical transformations represent fundamental biological mechanisms and critical technical factors governing silage quality. Additionally, the metabolic activities of LAB are critical for driving a rapid decline in pH during the ensiling process. This acidic environment effectively suppresses the proliferation of undesirable microorganisms, thereby preserving silage quality. This preservation mechanism is a well-documented factor in maintaining silage integrity and is extensively studied in academic research on forage conservation and animal nutrition science [6]. Pickling refers to a broad category of fermented vegetable products in which LAB is the primary microbial agent driving the fermentation process. Common LAB species include *Lactiplantibacillus plantarum*, *Lactiplantibacillus brevis*, and *Pediococcus acidilactici* [7]. The *Lactiplantibacillus* has been identified as the core microbial genus driving fermentation activity among these dominant bacterial populations. Despite current progress, several studies on silage-associated LAB and their functional components remain in the early stages, and their full potential has yet to be realized. Substantial research is required to elucidate the complex interactions among LAB, silage, livestock, and animal-derived products [4].

Interestingly, the composition of microbial communities in pickled foods has been shown to dynamically shift across different stages of fermentation, attracting extensive attention from researchers both domestically and internationally. Recent studies have reported successfully isolating and purifying 12 bacterial colonies from Gonggale pickles, classified within the genera *Weissella* and *Lactiplantibacillus* [7]. These isolated strains demonstrated strong viability under simulated gastrointestinal conditions and exhibited significant antimicrobial and antioxidant properties [8]. Taizhou pickle is a distinctive traditional fermented vegetable product native to China. Previous studies have shown that the key LAB involved in its fermentation is *Lactiplantibacillus clavatus*, which possesses the capability to suppress mold growth and reduce mycotoxin levels [9]. In the early fermentation, most pickles contain many *Leuconostoc mesenteroides*, *Lactiplantibacillus*, and *Weissella*. In contrast, the relative abundance of *Lactiplantibacillus brevis* showed a downward trend in the middle and late stages, while *Lactiplantibacillus plantarum* persisted throughout the entire fermentation process [10]. Some researchers say that pickles are a good source of probiotics and have beneficial health effects on the host.

Maize silage is a high-yield, starch-feed that provides energy for livestock [11]. The application of *Lactiplantibacillus plantarum* and *Lactiplantibacillus buchneri* as inoculants can influence the structure and dynamics of the bacterial community in whole-plant maize silage while enhancing its fermentation characteristics and stability during aerobic storage [12]. Moreover, maize silage is widely recognized for its high yield, rich nutrition, and excellent palatability. It is rich in fermentable WSC and is highly suitable for silage preparation. It serves as an important source of roughage in ruminant diets. The relatively high WSC content, in synergy with the naturally occurring LAB in the feed, facilitates an efficient and stable natural fermentation process. Due to their high content of high-quality protein and various essential vitamins, soybeans have become an indispensable and important protein feed resource in animal diets. However, their high protein concentration and strong buffering capacity can inhibit the growth of LAB, thereby interfering with the natural fermentation process. This inhibition may lead to poor silage fermentation, excessive BA production, and unpleasant odors. Compared to silage produced from single legume species, co-ensiling maize with leguminous crops results in a significant increase in lactic acid levels and a substantial reduction in AN content, while mixed silage exhibits lower AN concentrations than pure maize silage. However, compared to pure maize silage, the mixed silage shows lower AN concentrations. Thus, integrating leguminous crops with maize in silage preparation helps optimize the microbial community composition and improve fermentation performance [13]. Meanwhile, soybeans’ high protein content substantially enhances the feed’s nutritional quality [14]. In addition, it has been reported that grasses from the Poaceae family can alter the nitrogen-fixing capacity of leguminous forages [13].

Guizhou pickle is famous for its unique sour, spicy and crisp taste. Guizhou Province is the province with the widest daily distribution of rocky desertification temperature in Southwest China. This is due to the high population density, backward economy and unique climate, which also leads to the degradation of soil properties and microbial communities in these areas. The interaction of microorganisms, vegetation and soil, and different soil microbial communities in different regions lead to changes in the microbial community of pickled raw materials. The bacterial community composition in pickles has obvious regional specificity. The homemade pickles in the strong rocky desertification area show higher bacterial diversity but lower species richness. The differences in bacterial communities carried by pickled raw materials in different rocky desertification areas may be related to the correlation between soil bacterial diversity and rocky desertification areas [15]. Compared with high-temperature areas, family pickles in low-temperature areas showed lower bacterial diversity and species richness. This phenomenon can be attributed to the promotion of temperature increase on bacterial community diversity during fermentation [16]. The unique geographical environment in the Guizhou karst area may give pickles a unique flavor and nutritional value, and the rich microbial resources need to be further explored. Improving the quality of fermented vegetables can increase their nutritional value and provide health benefits.

Remarkably, traditional pickles from the karst region of Guizhou harbor diverse communities of LAB. These bacterial strains possess distinct metabolic characteristics and robust fermentation abilities, offering valuable natural microbial resources for developing novel fermentation starters. The present study successfully isolated three high-performing LAB strains from pickle samples, identifying them as *Lactiplantibacillus pentosus*, *Limosilactobacillus fermentum* and *Lactiplantibacillus plantarum*. The LAB derived from Guizhou pickles demonstrates both favorable probiotic attributes and consistent fermentation performance, indicating promising potential for application in silage production. Accordingly, we aim to investigate how these selected LAB influence the microbial composition and fermentation dynamics during the ensiling of whole-plant maize, whole-plant soybean, and their mixtures, and to identify effective strains suitable for silage feed additives. This study systematically analyzed the bacterial community and metabolite diversity of traditional pickles in the karst area of Guizhou for the first time, and found that they had significant geographical dependence. It not only fills the gap in the research field of microbial community and metabolites of traditional fermented food in karst areas but also provides a theoretical basis for the quality improvement and safety guarantee of traditional fermented food.

## 2. Materials and Methods

### 2.1. Isolation and Purification of LAB

In this study, the LAB were isolated and purified using a combination of gradient dilution and selective culture techniques. Briefly, 10 mL of pickle brine or 10 g of solid sample was suspended in 90 mL of sterile water. The suspension was vigorously shaken and subjected to cell disruption for 15 min. Subsequently, tenfold serial dilutions (10^−1^ to 10^−7^) were prepared. Four key dilutions (10^−1^, 10^−3^, 10^−5^ and 10^−7^) were selected, and 30 μL of each dilution was evenly spread onto the surface of de Man, Rogosa, and Sharpe (MRS) solid medium. The plates were incubated under anaerobic conditions in a 5%CO_2_ atmosphere at 37 °C for 24–48 h. Following incubation, single colonies exhibiting morphological characteristics indicative of LAB were selected and purified through 3–5 consecutive streak-plate isolations to obtain monoclonal strains with genetic uniformity. The purified strains were cultured in MRS liquid medium under shaking conditions (250 rpm, 37 °C) for 12–16 h. A 10^−6^ dilution was then prepared using a ten-fold serial dilution method, and 30 μL of this dilution was uniformly spread onto MRS solid medium. The plates were incubated anaerobically in a 5% CO_2_ atmosphere at 37 °C for 24–48 h. After single-colony formation, the isolates were subjected to preliminary identification through catalase testing and Gram staining. For long-term preservation, bacterial suspensions were mixed with a 50% glycerol solution at a 1:1 (*v*/*v*) ratio, transferred into sterile cryovials, and stored at −80 °C.

### 2.2. Characteristics of LAB Strains

**Figure 1:**
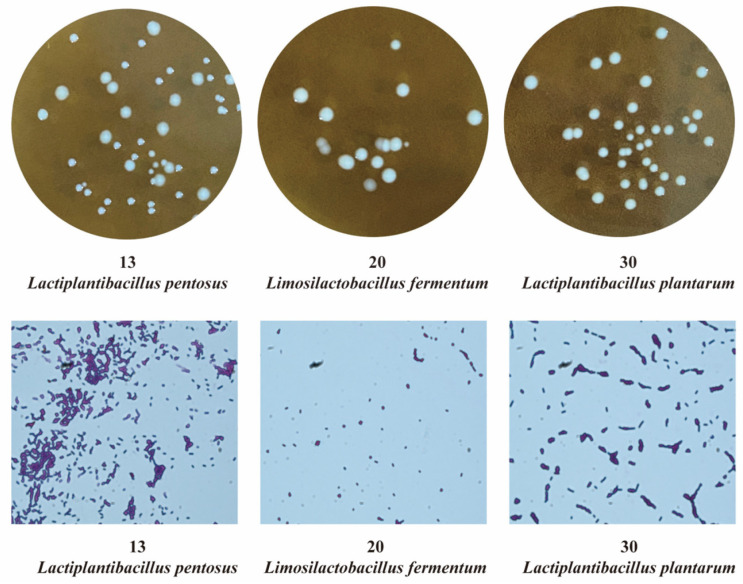
Phenotypic characteristics and Gram’s microscopic examination of LAB.

A total of 258 LAB strains were isolated from traditional pickle samples collected in Guizhou. Three high-performing candidate strains were selected through growth kinetics analysis, encompassing acid production and optical density (OD600) growth curves, and stress tolerance assessments (including resistance to acid, salt, and high temperatures). The phenotypic characteristics and Gram-stained images of these three superior LAB strains are presented in Figure 1, and their phenotypic traits and physiological properties are summarized in Table 1. Strain 13 formed round, milky-white, smooth, and glossy colonies, while strains 20 and 30 produced round, white, and smooth colonies. All three strains exhibited rod-shaped morphology, were Gram-positive (G^+^), and tested negative for catalase activity. These characteristics suggest that the isolates may be tentatively classified within the genus *Lactiplantibacillus*. According to Bergey’s Manual of Systematic Bacteriology, which provides detailed protocols for identifying and classifying LAB, the biochemical identification results for the selected LAB strains are presented in Table 2. Strain 13 does not liquefy gelatin, does not reduce nitrate, does not reduce acetate, produces hydrogen sulfide (H_2_S), can decompose gluconate, and is classified as a heterofermentative strain. Strain 20 does not liquefy gelatin, does not reduce nitrate, does not reduce acetate, does not produce H_2_S, does not decompose gluconate, and exhibits heterofermentative metabolism. Strain 30 does not produce H_2_S, does not reduce nitrate, does not reduce acetate, but can liquefy gelatin and decompose gluconate, and is characterized as a homofermentative strain.

**Table 1:** Phenotypic characteristics and physiological indexes of LAB.

Strain (Screening Number)	Source	Colony Characteristics	Cell Shape	Hydrogen Peroxide Activity	Gram Stain
13	Pickles (Guan Ling)	Milky white, round, smooth surface, glossy	bacilliform	−	+
20	Pickles (Pan Zhou)	White, round, smooth surface	bacilliform	−	+
30	Pickles (Ren Huai)	White, round, smooth surface	bacilliform	−	+

**Table 2:** Biochemical identification results of LAB.

Items	13	20	30
Hydrogen sulfide experiment	+	−	−
Gelatin liquefaction	−	−	+
Nitrate (reduction)	−	−	−
Acetate (reduction)	−	−	−
Gluconate	+	−	+
Glucose gas production	+	+	−
Glucose	+	+	+
Lactose	+	+	+
Galactose	+	+	+
Maltose	+	+	+
Sucrose	+	+	+
Fructose	+	+	+
Rhamnose	+	+	+
Arabinose	+	+	+
Xylose	+	+	+
Mannose	+	+	+
Melezitose	+	+	+
Raffinose	+	+	+
Inulin	+	+	+
Melibiose	+	+	+
Cellobiose	+	+	+
Starch	−	−	−
Sorbitol	+	+	+
Melampyrite	−	−	−
Mannitol	+	+	+
Inositol	−	−	−
Aesculin	+	+	+

“+” means positivity; “−” means feminine.

**Figure 2:**
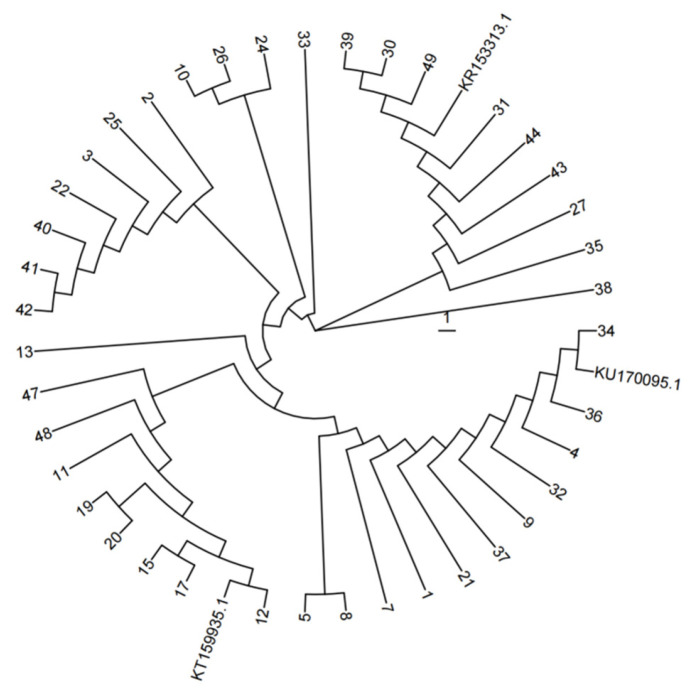
Phylogenetic tree analysis of LAB.

All three strains are capable of fermenting glucose, lactose, galactose, maltose, sucrose, fructose, rhamnose, arabinose, xylose, mannose, pine sugar, raffinose, inulin, melibiose, cellobiose, sorbitol, mannitol, and esculin but cannot ferment starch, eriodictyol, and inositol. Based on these biochemical characteristics, strain 13 can be further identified as either Lactobacillus pentosus or *Lactobacillus plantarum*; strain 30 as *Lactobacillus plantarum*; and strain 20 as *Lactobacillus mucilaginosus*. The three high-performing LAB strains were subjected to 16S rRNA gene sequencing, and the obtained sequences were compared against the NCBI (National Center for Biotechnology Information) database. The results are shown in Table 3. Strain 13 showed 100.00% sequence similarity to *Lactiplantibacillus pentosus*; strain 20 exhibited 99.86% similarity to *Limosilactobacillus fermentum*; and strain 30 displayed 99.93% similarity to *Lactiplantibacillus plantarum*. A phylogenetic tree was constructed based on the 16S rRNA gene sequences of LAB with rapid acid-producing ability, as illustrated in Figure 2. The accession numbers KU170095.1, KT159935.1 and KR153313.1 correspond to the reference strains of *Lactiplantibacillus pentosus*, *Limosilactobacillus fermentum*, and *Lactiplantibacillus plantarum*, respectively. Phylogenetic analysis revealed that strain 13 diverged by 11 branches from *Lactiplantibacillus pentosus*; strain 20 diverged by two branches from *Limosilactobacillus* fermentum; and strain 30 diverged by two branches from *Lactiplantibacillus plantarum*. Based on comprehensive biochemical and physiological characterization, strain 13 was identified as *Lactiplantibacillus pentosus*; strain 20 as *Limosilactobacillus* fermentum; and strain 30 as *Lactiplantibacillus plantarum*.

**Table 3:** Biological identification results of LAB.

Strain (Screening Number)	Reference Sequence	Results of Strain Identification	Similarity
13	KU170095.1	*Lactiplantibacillus pentosus*	100.00%
20	KT159935.1	*Limosilactobacillus fermentum*	99.86%
30	KR153313.1	*Lactiplantibacillus* *plantarum*	99.93%

All three strains demonstrated the ability to ferment glucose, lactose, galactose, maltose, sucrose, fructose, rhamnose, arabinose, xylose, mannose, melezitose, raffinose, inulin, melibiose, cellobiose, sorbitol, mannitol, and esculin. However, they could not ferment starch, eriodictyol, or inositol. Strains 13, 20, and 30 exhibited robust acid tolerance, salt tolerance, broad temperature adaptability, and significant diversity in sugar metabolism and efficient acid production capacity. Their primary function is to regulate microbial communities through rapid acidification. During fermentation, the strains entered the logarithmic growth phase within the first 4 to 14 h, preferentially utilizing soluble sugars in the environment and converting carbohydrates into lactic acid via the glycolytic pathway. This process reduced the system pH to below 4.0 within 24 h. The rapid acid production enhances the ecological competitiveness of these strains, effectively suppressing the growth of competing microorganisms, such as *Escherichia coli* and spoilage bacteria, by creating an acidic environment. This mechanism contributes to the biosafety of the fermentation process.

In summary, *Lactiplantibacillus pentosus*, *Limosilactobacillus* fermentum, and *Lactiplantibacillus plantarum*, three LAB strains isolated from Guizhou pickles, exhibit favorable probiotic and fermentation properties. These characteristics demonstrate their considerable potential for applications in silage modulation. Further investigations will evaluate the effects of these high-performing LAB strains on the fermentation quality of forage silage.

### 2.3. Preparation of Silage

The materials utilized in the silage experiment consisted of whole-plant maize harvested at the milking stage and whole-plant soybean collected during the seed-filling phase. These raw materials were harvested on *31 July 2024*, and provided by Yulong Ecological Beef Cattle Farm in Tuxi Town, Fenggang County, Guizhou Province. The LAB additives used in the silage test were *Lactiplantibacillus plantarum* (No. 30), *Lactiplantibacillus pentosus* (No. 13), and *Limosilactobacillus* fermentum (No. 20), which were screened from traditional pickles in Guizhou. Whole-plant maize and whole-plant soybeans were uniformly chopped to a length of 2–3 cm using a chaff cutter. Subsequently, experimental treatments were assigned into three groups: *Lactiplantibacillus plantarum* group, *Lactiplantibacillus pentosus* group, *Limosilactobacillus fermentum* group, and CK group (Non-bacterial inoculation). In addition, we also evaluated different silage substrates, including whole-plant maize silage alone (labeled as C), whole-plant soybean silage alone (labeled as S), and mixed silage of whole-plant maize and whole-plant soybean in a 1:1 ratio (labeled as M). After mixing three LAB strains, the mixture was applied to the raw materials at a rate of 2 mL per kilogram to ensure a final concentration of 10^5^ colony-forming units per gram. In the CK group, an equal volume of sterile distilled water (2 mL per kilogram) was applied under the same conditions. The experimental design included five sampling time points, and each treatment group consisted of three replicate samples of approximately 500 g, with a total of 180 samples [17], and replicates originated from independent silage units. The samples were placed in polyethylene vacuum packaging bags (30 cm × 40 cm), sealed with a vacuum sealer, and then fermented at 25 °C in the dark under anaerobic conditions. The samples were collected from each treatment group for analysis on the 1st, 3rd, 7th, 15th, and 30th days of storage.

### 2.4. Determination of Nutritional Quality of Silage

The standardized thermal drying method determined the silage’s dry matter (DM) content. Briefly, initial thermal inactivation was conducted at 120 °C, followed by gradient drying at 65 °C. The sample pretreatment followed the sequential deactivation, drying, and grinding protocol. Fresh forage and 30-day silage samples were placed in kraft paper bags, subjected to high-temperature inactivation at 120 °C for 30 min to terminate enzymatic activity, and then transferred to a constant-temperature oven at 65 °C for 48 h until achieving constant mass. Lastly, DM content was determined through precise weighing. The dried samples were further ground to a fine consistency to meet the requirements for subsequent analytical procedures.

### 2.5. Determination of Fermentation Quality of Silage

In this study, the metabolic characteristics of silage fermentation were revealed by time series dynamic monitoring. At key anaerobic fermentation time points (1, 3, 7, 15, and 30 days), 10 g of representative silage samples were collected for water-soluble metabolite extraction. The samples were diluted with sterile distilled water at a ratio of 1:9 (*w*/*v*) and homogenized. An ultrasonic pretreatment step was applied to disrupt cellular integrity for samples containing structural cell walls, such as soybean, followed by low-temperature extraction at 4 °C for 24 h. The extract was clarified using a dual-layer filtration system consisting of sterile gauze and filter paper, and its acidity was immediately measured using a pH meter. Subsequently, 5 mL aliquots of each filtered sample were transferred into cryotubes and stored at −80 °C for subsequent analysis of AN, lactic acid (LA), AA, BA, propionic acid (PA), and other organic acids via HPLC-based quantitative profiling. The concentration of organic acids was analyzed using Thermo (Vanquish Core) high-performance liquid chromatography (HPLC), while AN content was measured through the phenol-sodium hypochlorite colorimetric method.

### 2.6. Determination of Microorganism Quantity in Silage

Quantitative analyses of microbiota were performed using the agar plate counting method. Specifically, the silage samples (10 g) from various fermentation stages (1, 3, 7, 15, and 30 days) were mixed with 90 mL of sterile distilled water and subjected to constant-temperature shaking (37 °C, 150 rpm, 60 min) to create a homogeneous bacterial suspension. Subsequently, serial ten-fold dilutions ranging from 10^−1^ to 10^−7^ were prepared, and 10 μL of the diluted bacterial solution from four key dilution levels (10^−1^, 10^−3^, 10^−5^, and 10^−7^) was inoculated onto the surface of three types of selective media using a micropipette.

### 2.7. 16S rRNA Sequencing of Microbiota in the Silages

The study of silage microbiome needs to construct a standardized DNA genome analysis process, and its core includes DNA extraction, targeted amplification and high-throughput sequencing. Total genomic DNA was extracted using the cetyltrimethylammonium bromide (CTAB) method. Samples were rapidly flash-frozen in liquid nitrogen and then lysed. High-purity DNA was obtained (A260/A280 > 1.8). The V3-V4 region of the bacterial 16S rRNA gene was amplified using TransStart FastPfu DNA polymerase (TransGen Biotech, Shanghai, China) and primers 341F (5′-CCTACGGGNGGCWGCAG-3′) and 806R (5′-GGACTACHVGGGTWTCTAAT-3′). Amplified DNA products were verified by 1.5% agarose gel electrophoresis and purified using the QIAquick Gel Extraction Kit (QIAGEN), followed by pooling at equimolar concentrations. The sequencing library was prepared following the PacBio SMRTbell™ template preparation guidelines. The circular consensus sequencing of the full-length 16S rRNA gene was performed on the Sequel II platform using CCS mode. The raw sequence data were subjected to quality control filtering (read length > 1400 bp, Q30 quality score > 90%). The sequencing library was prepared following the PacBio SMRTbell™ template preparation guidelines. Finally, the species annotation was performed using the SILVA database (SSU rRNA v138), and bacterial taxonomic analysis was subsequently completed on the online bioinformatics platform (https://cloud.majorbio.com/page/project/overview, access on 25 February 2025). Technical services for microbial 16S rRNA sequencing were provided by Majorbio Biology Co., Ltd. (Shanghai, China).

### 2.8. Statistical Analysis

Two-factors analysis of variance was applied to evaluate the impact of Treatments (T), days of ensiling (D) and their interaction (T × D) on fermentation characteristics of whole-plant soybean silage, whole-plant maize silage, whole-plant maize and whole-plant soybean mixed silage and of treatment (T), materials (M) and their interaction (T × M) on chemical composition and aerobic stability of whole-plant maize and whole-plant soybean mixed silage. Data of changes in fermentation quality, nutritional quality, and bacterial community during days of ensiling were repeatedly compared with Duncan’s test, using the SPSS program version 27 (IBM Corp., Armonk, NY, USA). Differences were considered statistically significant only when the probability level was lower than 0.05 (*p* < 0.05).

## 3. Results and Discussion

### 3.1. Analyses of the Chemical Constituents of Fresh Soybean and Maize

Table 4 provides a detailed overview of the chemical composition and microbial counts present in the raw materials. Fresh soybean and maize’s DM content was 29.01% and 23.43% of fresh matter (FM), respectively. The crude protein (CP) content in fresh soybean and maize was found to be 22.08% and 9.11% of DM, respectively, surpassing the values reported in previous studies [18,19]. The WSC content and the corresponding LAB present in the raw material are critical factors that significantly influence the preservation quality of silage. Generally, for properly fermented silage, the recommended levels are 60–70 g kg^−1^ DM for WSC and a minimum of 5 log CFU g^−1^ FM for LAB [1,20]. In this study, the WSC content in fresh soybean was only 15.59 g kg^−1^ DM, below the threshold required for adequate fermentation. This indicates that direct ensiling of soybeans is prone to poor preservation, potentially promoting undesirable microbial proliferation and metabolic activity, thereby leading to substantial nutrient loss [18]. The quantities of attached LAB measured at 9.96 and 8.30 for the two materials satisfied the criteria for well-fermented silage.

### 3.2. Effects of LAB Addition on Fermentation Quality of Whole Plant Soybean and Whole Plant Maize and Their Mixture Silages

**Figure 3:**
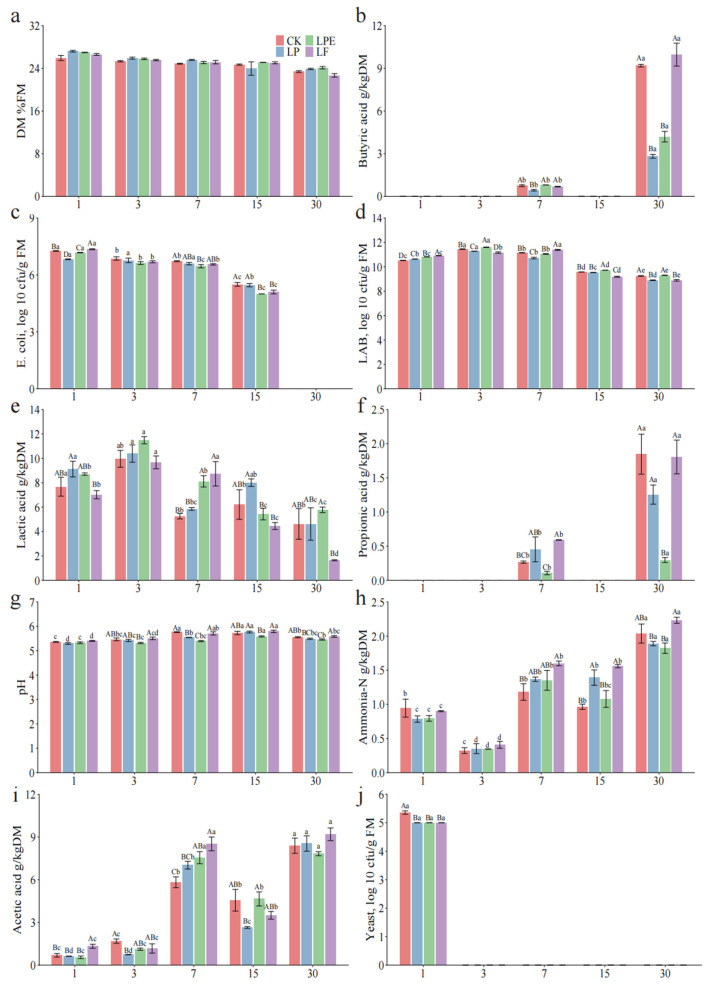
Effects of additives on fermentation characteristics of whole-plant soybean silage.

Figure 3 presents the effects of various treatments and fermentation stages on the fermentation characteristics of whole-plant soybean silage. A significant interaction (*p* < 0.05) was observed between additives and fermentation duration, influencing pH levels, concentrations of LA, AA, PA, BA, and AN, as well as the populations of LAB, yeast, and *Escherichia coli*. Additionally, the pH level and LA content are critical parameters for assessing silage quality. A pH below 4.2 is widely recognized as the threshold for high-quality silage [14,18]. However, after a 30-day ensiling period, the pH levels of all treatments exceeded 5.4. These findings suggest that the silage is inadequately preserved [21]. The rate of pH decline is considered a more critical indicator of silage quality than the final pH value [22]. In this study, after 7 days of ensiling, the pH levels of the *Lactiplantibacillus plantarum* (LP) and *Lactiplantibacillus pentosus* (LPE) treatments were significantly lower than those of the CK group (*p* < 0.05). Moreover, after 30 days of ensiling, the LPE treatment exhibited the lowest pH value (*p* < 0.05). These results indicate that both LP and LPE treatments facilitated a more rapid pH reduction in soybean silage. However, the LA concentration across all treatments decreased with prolonged ensiling duration, which contrasts with the findings reported by Mu et al. [22]. The primary explanation for these results may be attributed to the low WSC content and the high population of indigenous LAB in soybean silage (Table 4). Similarly, the WSC content is a critical limiting factor for fermentation, with a minimum requirement of approximately 30 g kg^−1^ DM to ensure successful silage fermentation [23]. In the present study, the WSC content (15.59 g kg^−1^ DM) was much lower than 30 g kg^−1^ DM. Therefore, the LAB lacked sufficient fermentation substrates for LA fermentation. The higher content of AA in silage is beneficial. The AA has antifungal properties. When exposed to air following the silage process, AA are crucial in inhibiting harmful microorganisms, including yeast [24].

Interestingly, after 1 day of ensiling, the AA content in the LF-treated silage was higher than in other treatments and remained the highest throughout the 30-day ensiling period. This phenomenon can be attributed to the incorporation of *Limosilactobacillus fermentum* (LF) treatment. This strain of heterofermentative LAB can simultaneously produce metabolites such as LA and AA [25]. Interestingly, despite adding homofermentative LAB in the LP and LPE treatments, AA content did not fall below that of the CK group throughout the fermentation process, except for LP-3d. This might be attributed to the relatively low WSC content in soybeans. Although inoculated with homofermentative LAB, it is usually converted to heterofermentative fermentation in grasses lacking WSC, especially legumes and tropical grasses [26]. The AA content increased with the prolongation of silage time and reached the highest after 30 days (*p* < 0.05), possibly due to the accumulation of LAB. On the other hand, this phenomenon may be attributed to the conversion of LA into AA by heterofermentative LAB during the later stages of silage fermentation. Due to the slow growth rate of heterofermentative LAB, converting LA into AA takes about 30–60 days to become obvious anaerobically [27]. The reduction in LA content over the duration of silage storage indirectly supports this assertion. A trace quantity of PA was observed across all treatments after 7 days and 30 days of silage fermentation. This phenomenon may arise from the conversion of LA into AA and 1,2-propanediol by heterofermentative LAB. Subsequently, 1,2-propanediol is transformed into PA [28]. After 30 days of silage, BA was observed in all treatment groups, aligning with the previous reported [29]. This observation could be attributed to the elevated pH levels across all treatments, which were insufficient to suppress the activity of *Clostridium*. Compared with the CK and LF groups, the levels of BA were significantly decreased in the LP and LPE groups (*p* < 0.05).

It is noteworthy that AN serves as a key indicator of CP degradation in silage, which typically arises from a gradual decline in pH levels or suboptimal microbial fermentation processes [30]. After 30 days of ensiling, the AN content in LP and LPE was significantly lower than that in CK and LF (*p* < 0.05), which may be due to their lower pH value inhibiting the activity of related protein-degrading microorganisms [18]. The AN content of all treatments increased with the extension of silage time (*p* < 0.05), which was consistent with the results of Mu et al. [22]. This phenomenon may be attributed to the consumption of protein by *Clostridium* during the later stages of silage fermentation, as evidenced by the rise in BA content during this period. This phenomenon can be attributed to the consumption of protein by *Clostridium* during the later stages of silage fermentation, as indicated by the increase in BA content during this period. Moreover, we observed that the population of LAB in the additive-treated silage was significantly greater than that in the CK group on the first day of silage fermentation (*p* < 0.05). It has been reported that silage inoculation with LAB substantially increases the LAB population within the silage [25]. As silage fermentation progressed, the population of LAB in all treatments declined significantly (*p* < 0.05). This reduction may be attributed to the limited availability of WSC in the later stages of fermentation, leading to the death of certain LAB strains due to reduced competitive ability. In addition, compared with the early stage of silage, the contents of AA, PA and BA in each treatment were higher in the late stage of silage, and AA, PA and BA had antibacterial properties [25].

**Figure 4:**
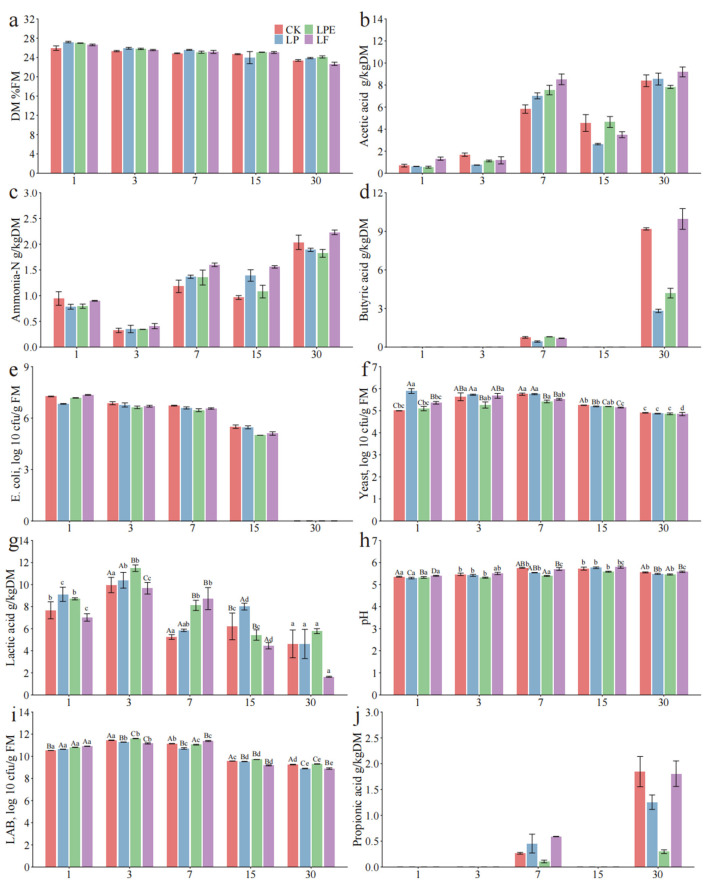
Effects of additives on fermentation characteristics of whole-plant maize silage.

The speed at which pH decreases during the ensiling process plays a crucial role in maintaining the nutritional quality of silage. Figure 4 illustrates the effects of different treatments and fermentation processes on whole plant maize silage quality, and the combined effect of additives and silage duration significantly influenced the pH level, LA content, and the populations of LAB and yeast (*p* < 0.05). After 30 days of silage fermentation, the pH levels of all treatments dropped below 4.2, reaching a threshold value that signifies successful silage preservation [21]. In this experiment, compared to the CK group, the additive-treated silage exhibited a significantly lower pH value on day 1 (*p* < 0.05), and this reduced pH level was sustained until day 30. Our analyses revealed that the additives effectively promoted a faster decline in silage pH. The LA content steadily increased as the silage fermentation progressed (*p* < 0.05), demonstrating a trend generally consistent with the variation in pH, except at the 15-day time point. LA plays a significant role in lowering the silage pH, and the acidity of this compound is over ten times greater than other organic acids, such as AA and PA [31]. The AA content rose gradually as the silage fermentation duration increased (*p* < 0.05), with no significant variation observed among the different treatments. This phenomenon may be attributed to the high initial population of LAB present on the raw materials and the ample availability of WSC, which potentially slowed down the growth rate of the added LAB strains [27]. Therefore, the silage fermentation process is predominantly characterized by homolactic fermentation.

On day 1 of silage fermentation, the application of additives resulted in a significantly higher LAB count compared to the CK (*p* < 0.05), which aligns with findings from previous research [25]. The observed reduction in pH of the additive-treated silage further substantiates this finding, indicating that LAB inoculation effectively facilitated a rapid decline in silage pH during the initial stages of fermentation. With the extension of silage time, the quantities of all treatments in the LAB decreased (*p* < 0.05), which may be due to inhibition of the activities of some low-acid-tolerant LAB, such as *Weissella*, *Enterococcus*, and most species in *Leuconostoc*, and the remaining LAB in the later stage of silage were mostly *Lactiplantibacillus* [32]. Throughout the entire fermentation period, no *Enterobacteriaceae* were detected. This phenomenon may primarily be attributed to the rapid establishment of an acidic environment due to the low pH in all treatments, which effectively suppressed the activity and growth of *Enterobacteriaceae* [18].

**Figure 5:**
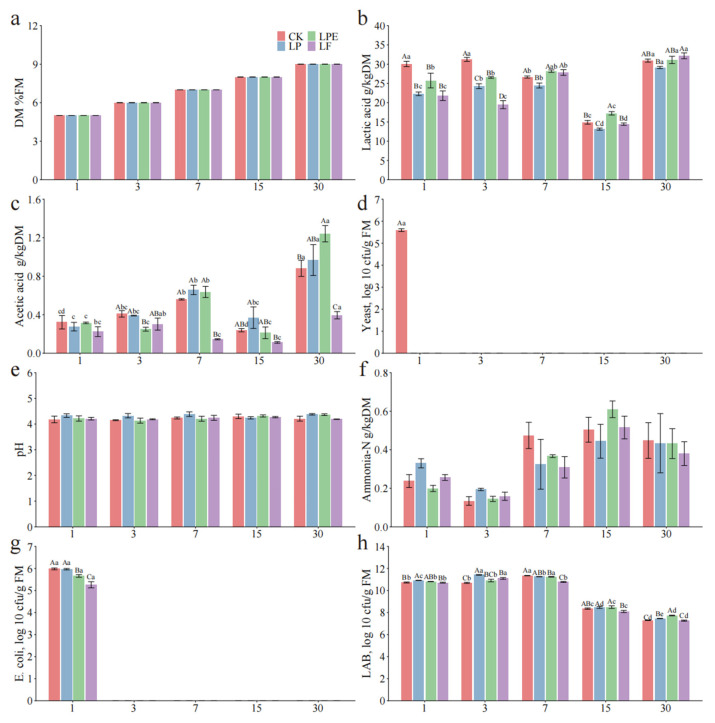
Fermentation characteristics of whole-plant maize and whole-plant soybean mixed silage.

Figure 5 illustrates the effects of different treatments and fermentation processes on the fermentation quality of mixed silage composed of whole-plant soybean and maize. The combined influence of silage duration and additive application had a significant impact on the concentrations of LA and AA, as well as the populations of LAB, yeast, and *Enterobacter* spp. species (*p* < 0.05). Throughout the entire silage process, the pH values across all treatments exhibited a relatively stable range, fluctuating between 4.13 and 4.38, with an average value of approximately 4.2. This stability may be primarily attributed to blending the two materials, wherein maize, characterized by a higher WSC content, effectively compensates for the sugar deficiency in soybeans. However, this might be attributed to the higher buffering capacity of soybeans, which made it more difficult for the pH to decrease [33]. After combining the two materials, the pH did not decrease to below 4.2 across all treatments. As the silage duration extended, the LA content in the additive-treated silage increased gradually (*p* < 0.05), a trend consistent with the findings reported by Mu et al. [22]. Following 30 days of ensiling, the differences in pH and LA content among the treatments became minimal. Additionally, the LAB count exceeded 5 lg CFU g^−1^ FM, indicating sufficient microbial activity to ensure successful silage fermentation [1]. This could be because the initial LAB count present on the raw material was already significantly higher than 5 l g CFU/g^−1^ FM (Table 4), suggesting that the supplementation of additional LAB had a limited impact. The AA content showed an increasing trend as the silage duration extended (*p* < 0.05), which might be attributed to the partial conversion of LA into AA during the fermentation process [25]. Similarly, the abundance of LAB declined as the silage duration increased (*p* < 0.05). We observed that the phenomenon may be attributed to the initially elevated levels of LAB during the early stages of fermentation, followed by the subsequent prevalence of acid-tolerant LAB species in later phases [32]. Yeast and *Enterobacteriaceae* were undetectable in the silage samples from day 3 to day 30, likely due to the inhibitory effects of the anaerobic and acidic conditions on their growth and metabolic activity [34].

### 3.3. Effects of LAB Addition on Nutritional Quality of Whole-Plant Soybean and Whole-Plant Maize and Their Mixture Silages

The interaction between additive application and mixing ratio significantly affected the contents of WSC, CP, neutral detergent fiber (NDF), acid detergent fiber (ADF), and aerobic stability (*p* < 0.05) (Table 5). As expected, combining soybean and maize in silage formulation resulted in a balanced nutrient profile, creating more favorable conditions for efficient fermentation. Maize contributed higher WSC levels, compensating for the deficiency in soybean, while soybean provided elevated CP content. Furthermore, aerobic stability was improved in mixed soybean–maize silage compared to maize silage alone. The incorporation of mixed silage facilitated the production of AA, which significantly inhibited the growth and metabolic activity of aerobic microorganisms, particularly yeasts. This process consequently improved the aerobic stability of the silage [25,26]. In the soybean-only silage treatments, additives effectively maintained the CP content compared to the CK group. This preservation effect is likely attributable to the lower pH levels observed in the LP and LPE treatments and the higher AA content in LF. These factors may inhibit plant protease activity and reduce protein degradation by limiting microbial activity [25]. In maize silage alone, the aerobic stability of the CK group was significantly lower (*p* < 0.05), with aerobic spoilage occurring after 18.33 h. This can likely be attributed to the high yeast populations in maize silage (Figure 4), as yeasts are a primary cause of aerobic deterioration in silage [35]. The application of additives significantly enhanced the aerobic stability of maize silage (*p* < 0.05), with the LF treatment exhibiting the most pronounced effect. This improvement is likely due to heterofermentative LAB in the LF treatment, which produces AA. This compound has been shown to inhibit aerobic microbial growth and metabolic activity, particularly yeasts [25].

### 3.4. Effects of LAB Addition on Bacterial Community of Whole-Plant Soybean, Whole-Plant Maize and Their Mixture Silages

The impact of additives on the bacterial diversity in whole-plant soybean silage is presented in Table 6. Across all samples, the average Goods Coverage exceeded 99%, indicating that the bacterial community analysis is reliable and feasible [18]. The ACE index was used to assess bacterial community richness, while the Shannon and Simpson indices were employed to evaluate bacterial community diversity [36]. The ACE and Shannon indices increased as the ensiling period progressed for the silage samples treated with additives (LP, LPE, and LF). In contrast, the Simpson index exhibited a corresponding decline over time. This indicates that the richness and diversity of the bacterial community in soybean silage diminished as the duration of ensiling increased. This decline may be attributed to the lower pH levels observed during the later stages of the silage process. Following 30 days of ensiling, the ACE and Shannon indices of soybean silage treated with LF were lower than those of other treatments, while the Simpson index was higher. This indicates that the LF treatment decreased the richness and diversity of the bacterial communities present. However, the pH level in the LF treatment was not significantly lower compared to other treatments (Figure 3). Therefore, this phenomenon might be attributed to the elevated AA content in the LF treatment, which suppressed microbial growth [37].

The influence of additives on the bacterial diversity in whole-plant maize silage is summarized in Table 7. The Goods Coverage exceeded 99% for all samples after ensiling, indicating that the results accurately reflect the actual condition of the samples [38]. In the CK group, both the ACE and Shannon indices exhibited an upward trend as the duration of silage increased, while the Simpson index displayed a downward pattern over time. In the LPE and LF treatments, the Shannon index declined while the Simpson index rose as the silage duration increased. After 30 days of silage fermentation, the additive treatment reduced the ACE and Shannon diversity indices in maize silage compared to the CK group, simultaneously leading to an increase in the Simpson index. These findings suggest that applying additives can significantly decrease the richness and diversity of bacterial communities present in maize silage. This could be attributed to the slower decline in pH observed in the CK group during ensiling. In contrast, the lower pH levels achieved in the additive-inoculated silage restricted microbial activity [22]. Furthermore, the impact of additives on the bacterial diversity in whole-plant soybean and maize mixed silage is presented in Table 8. Likewise, the Goods Coverage for all samples after ensiling exceeded 99%, demonstrating that the sequencing depth was sufficient to reflect the actual condition of the samples accurately [38]. Across all treatment groups, the ACE and Shannon indices showed an upward trend as the silage duration increased. In contrast, the Simpson index declined over time, with the CK group exhibiting a more pronounced increase. After 30 days of ensiling, the silage treated with LP demonstrated lower ACE and Shannon indices than other treatments, whereas the Simpson index was higher. The findings above indicate that additives can significantly diminish the richness and diversity of bacterial communities in mixed silage composed of soybean and maize, with the LP treatment exhibiting particularly pronounced effects.

**Figure 6:**
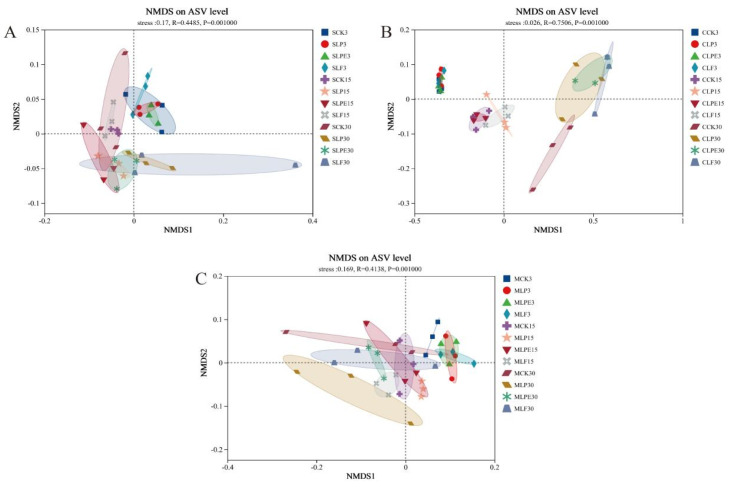
Non-metric multidimensional scaling (NMDS) analysis of bacterial community differences between different silage time and treatments. (**A**) whole plant soybean silage; (**B**) whole plant corn silage; (**C**) whole plant soybean and corn mixed silage.

Non-metric multidimensional scaling (NMDS) analysis further illustrated the clustering patterns of samples in soybean silage (Figure 6A). Following three days of ensiling, all sample points were predominantly clustered in the first quadrant. However, after 15 and 30 days of ensiling, the sample points were mainly distributed in the second, third, and fourth quadrants. This indicates that the bacterial community structure on day 3 differed significantly from that observed on days 15 and 30, with microbial composition becoming more stable after 15 days of ensiling. By day 30, the CK group was predominantly situated in the second quadrant, while the additive-treated samples were primarily clustered in the third and fourth quadrants. Previous research results suggest that the additives played a regulatory role in shaping the bacterial community structure of soybean silage [22]. Figure 6B illustrates the variations in bacterial community composition among maize silage samples. The sample points corresponding to different silage durations were clearly distinguishable. Among these, samples from 3 and 15 days of ensiling were primarily clustered in the second and third quadrants, respectively. In contrast, those from 30 days of ensiling were predominantly found in the first and fourth quadrants. This suggests that the bacterial community composition varied across the different ensiling stages [39]. Following 30 days of ensiling, the CK group was primarily clustered in the fourth quadrant, whereas those from the additive-treated groups were mainly located in the first quadrant. This observation implies the presence of distinct bacterial community structures between the CK and additive-treated silage samples [22]. Additives influenced the composition of the bacterial community in maize silage. Figure 6C illustrates the variations in bacterial community composition among soybean and maize mixed silage samples. Samples collected after 3 days of ensiling were predominantly clustered in the first quadrant, whereas those obtained after 30 days were primarily distributed across the second and fourth quadrants. This observation suggests distinct bacterial community structures between these two time points. Following 30 days of ensiling, the sample points from the LP treatment were primarily clustered in the third quadrant, distinctly separated from those of other treatments. This suggests that the LP treatment altered the bacterial community structure of the mixed silage.

**Figure 7:**
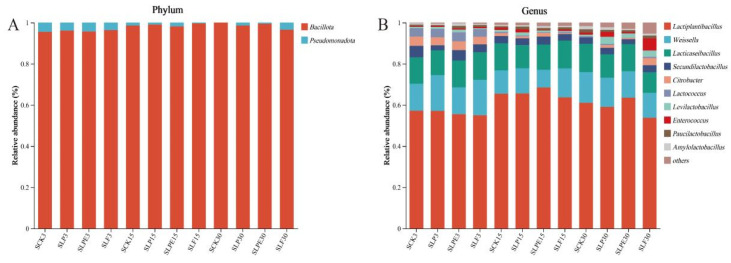
Bacterial community composition of soybean silage during ensiling process. (**A**) Phylum level; (**B**) Genus level.

The bacterial composition during soybean silage is shown in Figure 7. At the phylum level, the whole fermentation process comprises *Bacillota* and *Pseudomonadota*, of which *Bacillota* is the most dominant phylum. *Bacillota* is a microbial capable of acid hydrolysis, while *Pseudomonadota* is involved in the breakdown of organic matter and contributes significantly to nitrogen and carbon cycling during anaerobic digestion. Both are crucial for processes occurring in anaerobic environments [29]. *Bacillota* is the predominant phylum in silage, likely due to the anaerobic and acidic microenvironment, suppressing aerobic microorganisms and favoring the proliferation of LAB. The growth of *Bacillota* species is particularly supported under the low pH and oxygen-limited conditions typical of the silage process [40]. At the genus level, the primary bacterial genera present across all treatments during fermentation included *Lactiplantibacillus*, *Weissella*, *Lacticaseibacillus*, and *Secundilactobacillus*, all of which are classified under the broader genus *Lactiplantibacillus*. It is widely acknowledged that LAB plays a pivotal role in enhancing LA production and reducing pH levels, thereby establishing itself as a predominant genus in creating high-quality silage. In addition, we observed that the abundance of *Weissella* was higher in all treatment groups.

**Figure 8:**
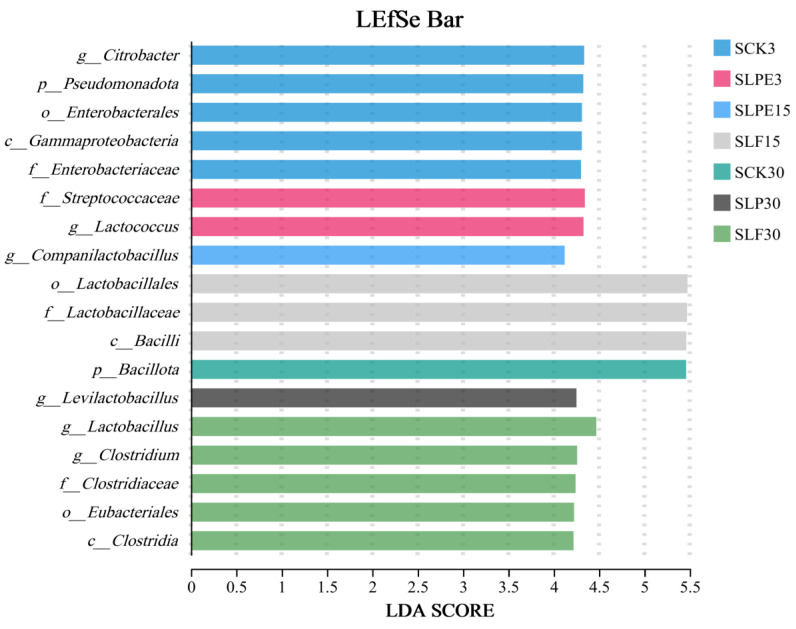
LEfSe analysis was used to analyze and compare the changes in the bacterial community across different treatments and soybean silage processes.

Notably, *Weissella* is generally regarded as an early colonizer, as it is typically outcompeted by acid-tolerant *Lactiplantibacillus* species during the progression of fermentation and the concomitant decline in pH [41]. It is a heterofermentative LAB that cannot thrive under low pH conditions [32]. Consequently, the elevated pH levels observed across all treatments contributed to their increased relative abundance. This observation also partly accounts for the elevated levels of AA across all experimental groups (Figure 3). As the silage duration increased, the relative abundance of *Citrobacter* in all treatments gradually declined. Microbial differences among the groups were further analyzed using linear discriminant effect size (LEfSe) (Figure 8), which revealed that *Citrobacter* was enriched explicitly in the SCK3 treatment. Citrobacter is a member of the *Enterobacteriaceae* family and is potentially detrimental to silage quality. The observed decline in its relative abundance may be linked to the emergence of an anaerobic environment during the later stages and the elevated levels of AA.

**Figure 9:**
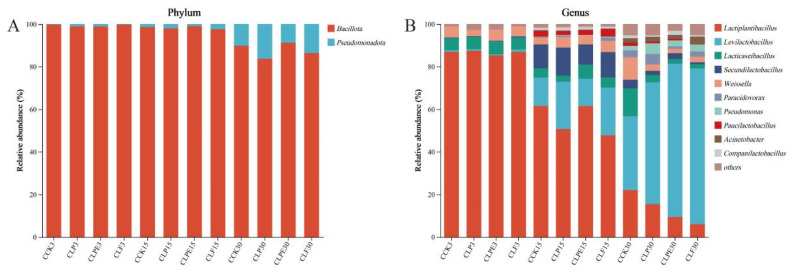
Bacterial community composition of corn silage during ensiling. (**A**) Phylum level; (**B**) Genus level.

**Figure 10:**
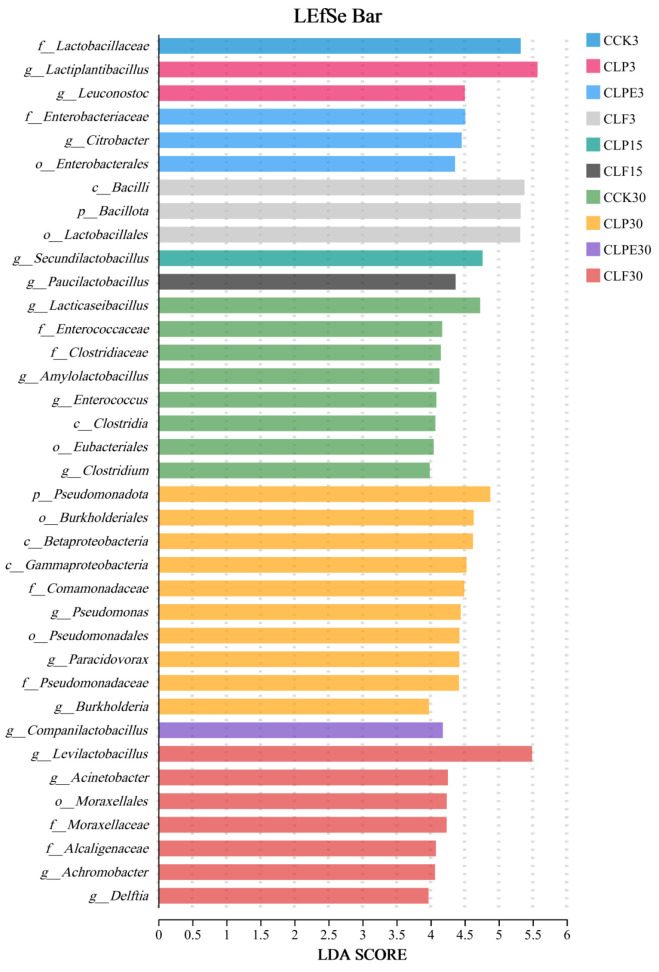
LEfSe analysis was used to compare the changes in the bacterial communities across different treatments and corn silage processes.

As shown in Figure 9, at the phylum level, *Bacillota* emerged as the predominant group across all treatments throughout the silage process. Following 30 days of silage fermentation, there was an increase in the relative abundance of *Pseudomonadota*, while the relative abundance of *Bacillota* declined. At the genus level, *Lactiplantibacillus* was the most dominant genus with short silage time after 3 and 15 days of silage. After 30 days of silage, *Levilactobacillus* became the dominant genus of each treatment, especially in the additive treatment, which was more obvious, similar to the previous research results [42]. *Levulinus* belongs to heterofermentative LAB. Heterofermentative LAB usually grow slowly. It usually takes 30–60 days to convert LA into AA in an anaerobic environment [25]. Therefore, this may be the reason for the high relative abundance of *Lactobacillus* after 30 days of silage. Meanwhile, it also partially explained the increase in AA content (Figure 4) in each treatment after 30 days of silage, and the improvement of aerobic stability by additive treatment. After 30 days of silage. The abundance of *Pseudomonas* was higher in each treatment, and the role of *Pseudomonas* in silage was still controversial. Previous studies have shown that *Pseudomonas* was negatively correlated with AN. Therefore, *Pseudomonas* may contribute to preserving proteins [43]. The LEfSe analyses (Figure 10) revealed that the bacteria of *Clostridium* were significantly enriched in the CCK30 treatment. This is undesirable in silage, as *Clostridium* produces metabolites such as BA, which reduces the nutritional quality of silage [44]. The application of additives effectively suppressed *Clostridium* growth, thereby improving silage quality.

**Figure 11:**
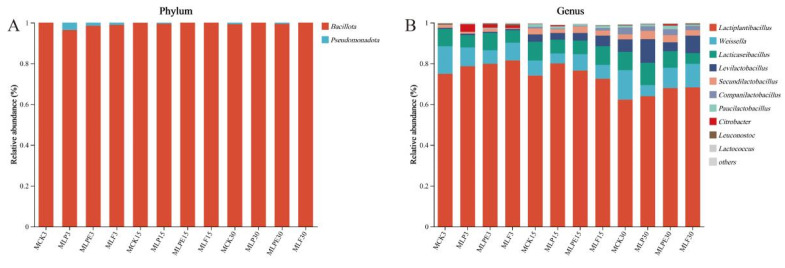
Bacterial community composition of soybean and corn mixed silage during ensiling. (**A**) Phylum level; (**B**) Genus level.

**Figure 12:**
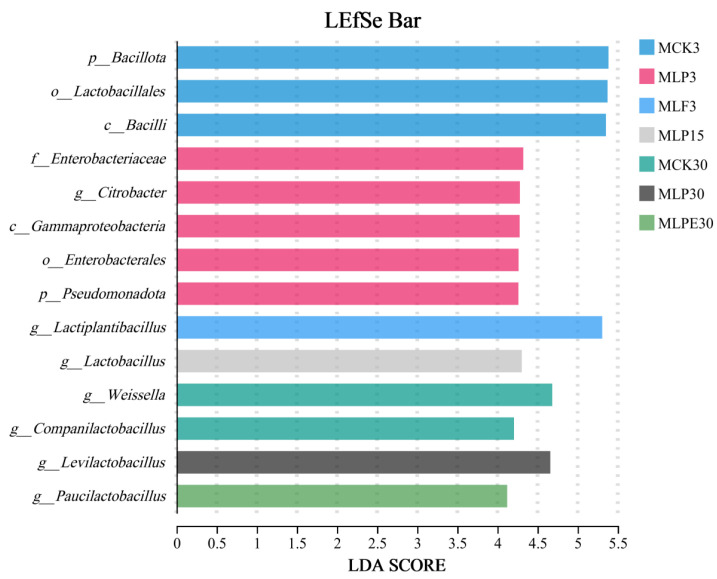
LEfSe analysis was used to compare the changes in the bacterial community across different soybean and corn mixed silage treatments.

The bacterial community composition during mixed silage of soybean and maize is shown in Figure 11. At the phylum level, *Bacillota* was the dominant phylum across all treatments throughout the ensiling process. At the genus level, the *Lactiplantibacillus* was the predominant genus in all treatments during ensiling. Additionally, the abundance of *Levilactobacillus* increased with prolonged ensiling duration, which may partially explain the observed increase in AA content across treatments after 30 days of fermentation. Additionally, the *Weissella* was detected in all treatments (Figure 4). Remarkably, this general *Weissella* typically exhibits poor acid tolerance [32]. The relatively low pH values (approximately 4.2) observed in each treatment may be attributed to the presence of acid-tolerant *Weissella* species. LEfSe analysis (Figure 12) indicated that *Weissella* was significantly enriched in the MCK30 treatment, likely due to the higher pH of this treatment.

## 4. Conclusions

Taken together, in this study, three high-performing LAB strains, *Lactiplantibacillus pentosus*, *Limosilactobacillus fermentum*, and *Lactiplantibacillus plantarum*, were isolated from traditional pickle samples in Guizhou Province. These strains exhibited exceptional adaptability to acidic (pH 4.0), saline (6% NaCl), and thermal (25 to 45 °C) conditions, along with efficient carbohydrate fermentation and rapid acid production. In soybean silage, *L. plantarum* and *L. pentosus* treatments lowered pH, AN, and BA while elevating lactic acid. Meanwhile, *L. fermentum* increased AA and reduced bacterial diversity; all strains preserved CP and altered community structure. For maize silage, they accelerated early pH reduction, conserved WSC, promoted late-stage *Levilactobacillus* growth for enhanced aerobic stability, and decreased *Clostridium* abundance. Because the CP content of soybean silage is relatively high, it can be charged to a higher level, and WSC content is low, so it is difficult to succeed in silage alone. In maize silage, the content of starch and WSC was higher, and the protein content was relatively lower. The strain addition boosted LAB abundance in mixed soybean–maize silage while suppressing yeast and *Escherichia coli* during initial fermentation. These findings demonstrate that LAB strains exert distinct and beneficial effects on silage quality, highlighting their practical potential in production.

## Figures and Tables

**Table 4:** Chemical composition and microbial quantity of soybean and maize before silage.

Items	Raw Materials	
	Soybean	Maize
pH	5.73	5.06
DM (% FM)	29.01	23.43
WSC (g kg^−1^ DM)	15.59	72.86
CP (% DM)	22.08	9.11
NDF (% DM)	43.75	52.10
ADF (% DM)	28.16	28.70
LAB (log_10_ CFU g^−1^ FM)	9.95	8.30
Yeast (log_10_ CFU g^−1^ FM)	5.46	5.57
*E. coli* (log_10_ CFU g^−1^ FM)	7.15	7.12

FM: fresh material; DM: dry matter; WSC: water-soluble carbohydrates; CP: crude protein; NDF: neutral detergent fiber; ADF: acid detergent fiber; LAB: lactic acid bacteria; CFU: colony-forming unit.

**Table 5:** Chemical composition and aerobic stability of whole-plant maize and whole-plant soybean mixed silage.

Items	Treatment	Materials			SEM	*p*-Value		
		S	C	M		T	M	T × M
WSC (g kg^−1^ DM)	CK	2.71 Cc	5.45 Db	6.72 Aa	0.34	<0.001	<0.001	<0.001
	LP	4.13 Ab	7.27 Ba	4.49 Bb				
	LPE	3.07 Bc	7.58 Aa	3.88 Cb				
	LF	0.66 Dc	6.89 Ca	4.47 Bb				
CP (%DM)	CK	18.49 Ba	10.62 c	16.72 BCb	0.63	0.003	<0.001	<0.001
	LP	19.15 Aa	10.67 c	17.46 Ab				
	LPE	19.42 Aa	10.17 c	16.48 Cb				
	LF	19.04 Aa	10.01 c	16.88 Bb				
NDF (%DM)	CK	38.20 BCb	52.32 Ba	40.04 Bb	1.03	0.002	<0.001	<0.001
	LP	36.90 Cc	54.89 Aa	38.34 Cb				
	LPE	39.68 ABc	52.60 Ba	41.29 Bb				
	LF	41.52 Ab	47.51 Ca	46.08 Aa				
ADF (%DM)	CK	27.01 Ca	27.46 BCa	24.95 BCb	0.36	<0.001	<0.001	<0.001
	LP	26.05 Db	31.98 Aa	24.16 Cc				
	LPE	28.10 Ba	28.42 Ba	25.45 Bb				
	LF	29.41 Aa	26.73 Cc	29.16 Ab				
Aerobic stability (h)	CK	144.00 a	18.33 Db	144.00 a	9.44	<0.001	<0.001	<0.001
	LP	144.00 a	24.33 Cb	144.00 a				
	LPE	144.00 a	28.67 Bb	144.00 a				
	LF	144.00 a	31.33 Ab	144.00 a				

Note: S, soybean; C, maize; M, soybean:maize (1:1); CK, no additives; LP, *Lactiplantibacillus plantarum* inoculant; LPE, *Lactiplantibacillus pentosus* inoculant; LF, *Limosilactobacillus fermentum* inoculant; A–D Means in the same column are different at level of *p* < 0.05; a–c Means in the same row are different at level of *p* < 0.05; ND, means not detected; T, means treatment; M, means materials; T × M, interaction between treatment and materials.

**Table 6:** Effects of additives on bacterial diversity of whole-plant soybean silage.

Items	Treatment	Fermentation Time		
		3	15	30
ACE	CK	41.23	44.01	42.09
	LP	47.84	41.12	43.52
	LPE	47.69	42.95	45.77
	LF	43.29	45.08	37.35
Shannon	CK	3.12	3.04	3.12
	LP	3.18	2.98	3.12
	LPE	3.28	2.99	3.11
	LF	3.16	3.11	3.05
Simpson	CK	0.064	0.077	0.067
	LP	0.063	0.081	0.071
	LPE	0.058	0.082	0.073
	LF	0.061	0.069	0.070
Goods Coverage	CK	0.997	0.996	0.998
	LP	0.996	0.997	0.997
	LPE	0.995	0.995	0.998
	LF	0.998	0.997	0.998

Note: CK, no additives; LP, Lactiplantibacillus plantarum inoculant; LPE, Lactiplantibacillus pentosus inoculant; LF, Limosilactobacillus fermentum inoculant.

**Table 7:** Effects of additives on bacterial diversity of whole-plant maize silage.

Items	Treatment	Fermentation Time		
		3	15	30
ACE	CK	30.74	32.56	53.76
	LP	26.45	32.59	35.14
	LPE	30.23	31.29	31.13
	LF	29.86	32.16	31.70
Shannon	CK	2.45	2.83	3.28
	LP	2.39	2.79	2.56
	LPE	2.44	2.83	2.25
	LF	2.46	2.91	2.12
Simpson	CK	0.135	0.083	0.072
	LP	0.133	0.085	0.159
	LPE	0.133	0.085	0.208
	LF	0.129	0.075	0.242
Goods Coverage	CK	0.998	0.998	0.994
	LP	0.998	0.996	0.998
	LPE	0.998	0.998	0.996
	LF	0.998	0.999	0.996

Note: CK, no additives; LP, Lactiplantibacillus plantarum inoculant; LPE, Lactiplantibacillus pentosus inoculant; LF, Limosilactobacillus fermentum inoculant.

**Table 8:** Effects of additives on bacterial diversity in whole-plant soybean and maize mixed silages.

Items	Treatment	Fermentation Time		
		3	15	30
ACE	CK	32.39	41.33	39.12
	LP	35.91	40.21	31.39
	LPE	39.81	41.04	40.28
	LF	35.46	41.57	37.39
Shannon	CK	2.70	2.89	3.03
	LP	2.70	2.69	2.81
	LPE	2.81	2.87	2.98
	LF	2.68	2.89	2.91
Simpson	CK	0.107	0.090	0.076
	LP	0.105	0.113	0.088
	LPE	0.098	0.092	0.081
	LF	0.112	0.091	0.084
Goods Coverage	CK	0.999	0.997	0.999
	LP	0.997	0.993	0.999
	LPE	0.996	0.996	0.997
	LF	0.997	0.996	0.999

Note: CK, no additives; LP, Lactiplantibacillus plantarum inoculant; LPE, Lactiplantibacillus pentosus inoculant; LF, Limosilactobacillus fermentum inoculant.

## Data Availability

Raw data have been deposited to National Center for Biotechnology Information (NCBI) under the BioProject number PRJNA1411022.
